# RETRACTED: Toncheva et al. Pathogenic Variants Associated with Rare Monogenic Diseases Established in Ancient Neanderthal and Denisovan Genome-Wide Data. *Genes* 2023, *14*, 727

**DOI:** 10.3390/genes16091026

**Published:** 2025-08-29

**Authors:** Draga Toncheva, Maria Marinova, Todor Chobanov, Dimitar Serbezov

**Affiliations:** 1 Medical Faculty, Department of Medical Genetics, Medical University of Sofia, Sofia 1000, Bulgaria; d.serbezov@medfac.mu-sofia.bg; 2 Bulgarian Academy of Sciences, Sofia 1000, Bulgaria; 3 Department of Computer Systems and Technologies, Faculty of Electronics and Automation, Technical University of Sofia, Branch Plovdiv, Plovdiv 4000, Bulgaria; m_marinova@tu-plovdiv.bg; 4 Institute of Balkan Studies, Centre of Tracology at the Bulgarian Academy of Sciences, Sofia 1000, Bulgaria; chobanov@abv.bg

The journal retracts the article “Pathogenic Variants Associated with Rare Monogenic Diseases Established in Ancient Neanderthal and Denisovan Genome-Wide Data” [1], cited above.

Following publication, concerns were raised with the publisher regarding the methodology and interpretation of the data, specifically relating to the accuracy of the genotypic analysis and the validity of the drawn conclusions.

Adhering to our procedure, an investigation was conducted by the Editorial Office and Editorial Board, which confirmed the fundamental flaws in the analysis and the unsupported nature of the findings. As a result, the Editorial Board has lost confidence in the integrity of the findings and decided to retract this publication [1], as per MDPI’s retraction (https://www.mdpi.com/ethics#_bookmark30, accessed on 8 July 2025).

This retraction was approved by the Editor-in-Chief of the *Genes* journal.

The authors did not comment on this retraction.
